# Abnormal adipogenic signaling in the bone marrow mesenchymal stem cells contributes to supportive microenvironment for leukemia development

**DOI:** 10.1186/s12964-023-01231-z

**Published:** 2023-10-10

**Authors:** Rawan Sabbah, Sahar Saadi, Tal Shahar-Gabay, Shiran Gerassy, Shlomit Yehudai-Resheff, Tsila Zuckerman

**Affiliations:** 1 Clinical Research Institute at Rambam, Rambam Health Care Campus, 3109601 Haifa, Israel; 2 The Ruth and Bruce Rappaport Faculty of Medicine, 3109601 Technion, Haifa Israel; 3 Department of Hematology and Bone Marrow Transplantation, Rambam Health Care Campus, 8, Ha’Aliya Street, 3109601 Haifa, Israel

**Keywords:** Mesenchymal stem cells, Bone marrow niche, Acute myeloid leukemia, Adipocytes, Osteocytes

## Abstract

**Background:**

Acute myeloid leukemia (AML) is an aggressive hematological malignancy, associated with unfavorable patient outcome, primarily due to disease relapse. Mesenchymal stem cells (MSCs) residing in the bone marrow (BM) niche are the source of mesenchyma-derived subpopulations, including adipocytes, and osteocytes, that are critical for normal hematopoiesis. This study aimed to characterize BM-derived adipocyte/osteocyte fractions and their crosstalk with AML cells as a potential mechanism underlying leukemogenesis.

**Methods:**

BM cell subpopulations derived from primary AML patients were evaluated using humanized ex-vivo and in-vivo models, established for this study. The models comprised AML blasts, normal hematopoietic stem and progenitor cells and mesenchymal stromal subpopulations. ELISA, FACS analysis, colony forming unit assay, whole exome sequencing and real-time qPCR were employed to assess the differentiation capacity, genetic status, gene expression and function of these cell fractions. To explore communication pathways between AML cells and BM subpopulations, levels of signaling mediators, including cytokines and chemokines, were measured using the ProcartaPlex multiplex immunoassay.

**Results:**

The study revealed deficiencies in adipogenic/osteogenic differentiation of BM-MSCs derived from AML patients, with adipocytes directly promoting survival and clonogenicity of AML cells in-vitro. In whole exome sequencing of BM-MSC/stromal cells, the *AHNAK2* gene, associated with the stimulation of adipocyte differentiation, was found to be mutated and significantly under-expressed, implying its abnormal function in AML. The evaluation of communication pathways between AML cells and BM subpopulations demonstrated pronounced alterations in the crosstalk between these cell fractions. This was reflected by significantly elevated levels of signaling mediators cytokines/chemokines, in AML-induced adipocytes/osteocytes compared to non-induced MSCs, indicating abnormal hematopoiesis. Furthermore, in-vivo experiments using a fully humanized 3D scaffold model, showed that AML-induced adipocytes were the dominant component of the tumor microenvironment, providing preferential support to leukemia cell survival and proliferation.

**Conclusions:**

This study has disclosed direct contribution of impaired functional, genetic and molecular properties of AML patient-derived adipocytes to effective protection of AML blasts from apoptosis and to stimulation of their growth in vitro and in vivo, which overall leads to disease propagation and relapse. The detected AHNAK2 gene mutations in AML-MSCs point to their involvement in the mechanism underlying abnormal adipogenesis.

Video Abstract

**Supplementary Information:**

The online version contains supplementary material available at 10.1186/s12964-023-01231-z.

## Background

Acute myeloid leukemia (AML) is the most prevalent leukemia type diagnosed in adults, with the estimated rate of new cases amounting to 4.1 per 100,000 cases per year and the death rate of 2.7 per 100,000 cases per year in the United States. Despite significant advances in the diagnosis and treatment of this aggressive hematological malignancy, the ratio between the number of new cases and the death rate has remained consistent over the last two decades [1]. Such mortality is mainly attributed to a high rate of disease relapse, which renders crucial the elucidation of its initiating mechanisms.

Hematopoietic stem and progenitor cells (HSPCs), inhabiting the bone marrow (BM) niche, are supported by mesenchymal stromal cells [2]. The latter cells give rise to the majority of marrow stromal cell lineages, including chondrocytes, osteoblasts, fibroblasts, adipocytes, endothelial cells and myocytes [3–6]. The crosstalk of these BM niche components with HSPCs is critical for normal hematopoiesis [7, 8] and is reported to be impaired in hematological malignancies, including leukemia [9–12]. It is suggested that reprogramming of mesenchymal stromal cells towards increased adipocyte differentiation affects normal hematopoiesis, leading to the creation of the microenvironment (ME) favorable for leukemia development [13]. However, the functional role of specific niche components in the initiation and propagation of AML remains debatable [14, 15]. It is unclear whether these alterations originate from the niche itself or from the crosstalk with leukemic cells. Adipocytes and osteocytes are recognized as the major contributors to the BM-ME; yet, data regarding their differentiation potential are contradictory. Some reports suggest an increased osteogenic potential [16], whereas others show an increased adipogenic potential [13]. Moreover, mesenchymal cells derived from AML patients demonstrate adipogenic and osteogenic differentiation deficiencies, changing the composition of the cellular niche [17, 18]. The genetic make-up and molecular mechanisms governing these aberrations are not fully elucidated. The *AHNAK2* gene, belonging to the nucleoprotein AHNAK family, is reported to be associated with the stimulation of adipocyte differentiation [19, 20], through shuttling between the cell nucleus and the cytoplasm*.* Moreover, AHNAK2 is suggested to regulate adipogenesis by signaling smad1-related PPARγ [21]. Additionally, this protein plays a role in calcium signaling and cytoarchitecture [22], which is of importance, given that the BM niche-calcium homeostasis is suggested to be crucial for leukemia initiation [23].

Due to the established critical role of BM mesenchyma-derived subpopulations in normal hematopoiesis, the current study investigated a potential impact of abnormalities in adipocytes and osteocytes on AML progression. We identified aberrations in adipogenic/osteogenic differentiation of BM mesenchymal stem cells (BM-MSCs) of AML patients. Importantly, AML-induced adipocytes were found to provide direct preferential support to leukemia cell survival, proliferation and clonogenicity both in-vitro and in-vivo, as demonstrated using a fully humanized 3D scaffold model. Furthermore, mutated AHNAK2 gene, known to be involved in adipocyte differentiation, was detected in AML-derived BM mesenchymal stromal cells. Overall, this study revealed a direct contribution of impaired functional, genetic and molecular properties of AML-derived adipocytes to leukemia propagation.

## Methods

### Human samples

This study was approved by the Institutional Review Board of the Rambam Health Care Campus (Approval #0023–15). Informed consent was obtained from all study participants. Fresh BM samples were collected at diagnosis from 26 primary AML patients (13 males, 13 females; median age 54 ± 18 years), who attended the Rambam Department of Hematology. BM samples of 26 age-matched healthy donors (HD; 10 males, 16 females; median age 50 ± 18 years), who underwent hip replacement surgery, were used as a control. For each sample, plasma was separated from the BM and was immediately frozen at -80 °C. A cell line of normal human BM derived MSCs (Lonza, Haifa, Israel, cat. #PT-2501) was used in calibration experiments and as a mesenchymal cell control. The OCIAML3 cell line, carrying DNMT3A, R882C and NPM1 driver mutations, was used as an AML cell control. The main clinical and biological parameters of each patient included in the study are shown in Tables S1, S2.

### Isolation and expansion of BM mesenchymal stem/stromal cell fractions

For cell preparation, mononuclear cells (MNC) were isolated from BM aspirates using density gradient centrifugation with LymphoPrep™ (PROGEN Biotechnik, cat. #1,114,547, Heidelberg, Germany).

To expand the mesenchymal stromal cell fraction, isolated MNCs were cultured in the Mesencult medium [Mesencult™ Proliferation Kit (human), STEMCELL Technologies Inc, cat. #05411, Vancouver, BC, Canada], with an addition of 10% AB human serum and 1% penicillin–streptomycin solution. BM stromal cells were generated up to passage (P) 3. To enrich and expand the MSC fraction, the isolated MNCs were cultured in the starvation medium RPMI (Biological Industries, cat. #1640, Beit-Haemek, Israel), with an addition of 10% FBS/human serum and 1% penicillin–streptomycin solution.

To induce MSC differentiation into adipocytes/osteocytes, BM-MSCs were stimulated using adipogenic or osteogenic differentiation inducing factors. In brief, P0 MSCs were cultured for 21 days at 37^0^C in either adipogenic or osteogenic medium (basal medium: MEM-α Biological Industries, cat. #01–042-1A; adipogenic supplement: R&D Systems, cat. #CCM011, Minneapolis, MN, USA; osteogenic supplement: R&D Systems, cat. #CCM008). The obtained cells are further referred to as “induced” MSCs, adipocytes or osteocytes. The MSCs that have not undergone such stimulation are further termed “non-induced”.

### ELISA assay

To estimate the status of adipogenesis and osteogenesis in samples derived from AML patients, secreted levels of the biomarkers human fatty-acid-binding protein 4 (FABP4; R&D Systems, cat. #DFBP40) and human osteocalcin (R&D Systems, cat. #DSTCNO) were measured in originally collected, frozen and thawed BM plasma (BMP) samples, obtained from HDs and AML patients at diagnosis. The sensitivity of the ELISA kits was 38 pg/mL for FABP4 and 0.898 ng/mL for osteocalcin.

### Flow cytometry analysis of the differentiation potential of induced adipocytes and osteocytes

The flow cytometry technique was applied to distinguish between MSCs (CD73 + and CD90 +) and AML cells (CD45 + and CD34 +).

Induced adipocytes or osteocytes were trypsinized, fixated with 2% or 4% paraformaldehyde for 10 min. at the room temperature (RT), and permeabilized with saponin 0.2% or triton 0.1% for 15 min. Non-induced MSCs and induced adipocytes or osteocytes were stained with primary antibodies: anti-human FABP4 (Thermo Fisher Scientific, cat. #710,189, Waltham, MA, USA) or anti-human alkaline phosphatase (ALP) (BioLegend, cat. #358,702, San Diego, CA, USA), respectively, and incubated for 30 min. (Table S3). Secondary antibodies (Thermo Fisher Scientific, cat. #F2765, BioLegend #405,308) were incubated for another 30 min. at RT in darkness. The cells were evaluated using the LSRFortessa cell analyzer (BD Biosciences, San Jose, CA). Analyses were performed using FlowJo V10 software.

### The “patient-in-a-dish” system

To mimic the AML-ME and investigate a crosstalk between BM-derived adipocyte and/or osteocyte subpopulations and leukemic cells, a “patient-in-a-dish” ex-vivo system was generated. Leukemic cells were sorted using FACS (Aria II; BD Biosciences) based on the leukemia-associated immunophenotype (LAIP), specific for each patient. The MNCs isolated from whole cord blood (CB) samples were employed as a control and were further enriched for CD34 + progenitor cells, using magnetic CD34 MicroBead Kit (human; Miltenyi Biotec, cat. #130,046–702, Bergisch Gladbach, Germany).

AML- or HD-induced adipocytes/osteocytes were then cultured in the MEM-α. CD34 + cells derived from either leukemic or CB cells were co-cultured (for 10 days) with the above MSC feeder layer at a density of 2 × 10^4^ cells/cm^2^ in the MyeloCult™ H5100 medium (STEMCELL Technologies Inc, cat. #H5100) with the addition of 1% penicillin–streptomycin solution. Cells were trypsinized, harvested and sorted for CD45 + cells, using CD45 Microbeads (human; Miltenyi Biotec, cat. #130–045-801) for simultaneous conduction of the colony forming unit (CFU) assay. The cell viability was assessed using the flow cytometry analysis.

### Colony forming unit assay

Sorted CD45 + cells were cultured with Methocult H4435 medium (STEMCELL Technologies Inc, cat. #04435, Vancouver, BC, Canada) for 14 days. The formed colonies were counted and classified based on their morphology. The quantification analysis was normalized to the initial number of seeded leukemic or CB cells (20,000 leukemic cells and 500 cells of CB CD34 +) and compared to the corresponding HD CFU results.

### Cell viability assay

Sorted CD45 + cells were collected and stained with Annexin V-FITC (BioLegend, cat. #640,906) and propidium iodide (PI). Subsequently, early and late apoptosis analyses were performed with the FITC signal detector (FL1) and the PI signal detector (FL2) using LSRFortessa. The data were analyzed with FlowJo V10 software. The experiments were conducted in triplets, irrespective of result similarity.

### ProcartaPlex multiplex immunoassay for cytokine screening

The crosstalk between AML cells or HD hematopoietic cells and the BM niche cells was evaluated by measuring secreted protein levels in the conditioning medium collected from the co-cultures.

The measurement was performed using the Invitrogen™ ProcartaPlex™ Human 13-plex panel (Thermo Fisher Scientific, cat. #EPX650-10,065–901). The cell culture supernatant was collected and frozen for further probing with the following markers: GM-CSF, IFN-ɣ, IL-6, IL-8, IL-10, MCP-1, SDF-1α, TNF-α, IDO, SCF, TPO, OPN, ANG1.

### Fully humanized scaffold model

The study was approved by the Technion Ethics Committee for Animal Research (Approval #IL0750521). A versatile humanized BM microenvironment system was used to mimic the human physiological and pathological conditions in an immunodeficient mouse model, allowing to explore normal and malignant human hematopoiesis. The experiments were performed as previously described [24, 25]. However, certain modifications were done, given that the main purpose of the current study was to explore a potential ability of major BM cell subpopulations and primary leukemic cells, both exclusively derived from AML patients, to support leukemia propagation. To address the well-known challenges associated with the generation of such fully humanized structure, two sets of experiments were concurrently conducted with either AML patient-derived MSCs co-cultured with the OCIAML3 cell line or patient-derived leukemic CD34 + cells co-cultured with a normal human MSC cell line; each co-culture was then seeded on 3D scaffolds. Notably, in these setups the two aforementioned cell lines were used to support the growth of patient-derived cells. Briefly, MSCs derived from 4 AML patients or a normal human MSC cell line (non-induced MSCs and 50% induced adipocytes/osteocytes) were re-suspended in MEM-α with 10% FBS and 50 µl of the suspension (1 × 10^5^ cells) were added to the scaffolds, that were then cultured in vitro for 3–5 days to expand cell fractions. Patient-derived leukemic CD34 + cells (1 × 10^5^ to 4 × 10^5^ cells) or the OCIAML3 cell line (1 × 10^5^ cells) were added to the pre-coated scaffolds. The medium was then replaced with MyeloCult™ H5100 (STEMCELL Technologies Inc, cat #05100, Vancouver, BC, Canada). Scaffolds with seeded cells were incubated for another 2 days at 37^0^C, and then were subcutaneously implanted to non-conditioned NSG mice (maximum 4 scaffolds/mouse). Twenty-eight days post-implantation, the mice were sacrificed, the scaffolds were retrieved and scaffold-derived tumors were extracted for weighing and fixation in 4% paraformaldehyde for further investigation. HD-MSCs were used as a control.

### Adipocyte staining and analyses

The density of adipocytes in hematoxylin–eosin (H&E) stained sections of the scaffold-derived tumors was estimated using ImageJ software [26].

For histological characterization and evaluation of morphological integrity, 5-µm-thick sections were stained with the Harris-modified, hematoxylin solution and eosin (Pioneer Research Chemicals Ltd, cat. #PRC/R51 and #PRC/66/1, respectively, Colchester, UK). Mouse-derived human adipocytes were identified in the tumor using the pan-leukocyte marker CD45 (eBioscience, cat. #14–0451-82, San Diego, CA) and the mouse adipocyte marker CD34 (BioLegend, cat. #119,303).

### FABP4 immunofluorescence staining

Cells were fixed with 4% paraformaldehyde for 15 min. at RT and permeabilized with 0.3% Triton X-100. After blocking, slides were incubated with an anti-human FABP4 polyclonal antibody (Thermo Fisher Scientific, cat. #710,189, Waltham, MA, USA) followed by incubation with a secondary antibody (1:1000, Jackson ImmunoResearch Laboratories, cat. AB_2338052). Images of live cells were captured with a confocal microscope (Nikon). Analysis and quantification of expression rates were performed using the FIJI software.

### AHNAK2 immunofluorescence staining

Cells were fixed with 4% paraformaldehyde for 15 min. at RT, and permeabilized with 0.3% Triton X-100. After blocking, slides were incubated with the AHNAK2 antibody (1:200, Sigma-Aldrich, cat. #HPA000878, St. Louis, MO, USA) followed by incubation with secondary antibodies (1:2000, Thermo Fisher Scientific, cat. #F2765) and ActinRed™ (Thermo Fisher Scientific, cat. #R37112). Images of live cells were captured with a confocal microscope (Nikon). Analysis and quantification of expression rates were performed using the IMARIS software.

### Whole exome sequencing

Whole exome sequencing was performed using the Illumina HiSeq 2500 platform. Genomic DNA was extracted from stromal cells (P3) and germline (saliva) using the DNA purification kit (Norgen Biotek, cat. #53,100, Thorold, ON, Canada). gDNA (2 µg) was employed for target enrichment using the Illumina TrueSeq exome kit and for single nucleotide variation analysis. The information regarding the detected genetic alterations is presented in Table S4. A mutational heat-map was generated using the R-software, version R-3.6.0 (ComplexHeatmap package).

### Real-time quantitative polymerase chain reaction (qPCR)

RNA was extracted from mesenchymal cells using the Total RNA Purification Micro Kit (Norgen Biotek, cat. #35,300). cDNA was synthesized using the qPCRBIO cDNA Synthesis kit (PCR Biosystems Ltd, cat. #PB30.11–10, London, UK). qPCR was performed using Absolute Blue qPCR SYBR Green ROX Mix (Thermo Fisher Scientific, cat. #AB-4163/A) with 10 mM of forward and reverse primers (Table S5). The qPCR program included: 15 min. at 95^0^C followed by 40 consecutive cycles of 15 s at 95^0^C and 20 s at 60^0^C. The QuantStudio™ Design & Analysis Software1.3.1 (Applied Biosystems, Waltham, MA, US) was used.

### Statistical analysis

The following statistical tests were performed: the Mann–Whitney test, an unpaired t-test and repeated-measures analysis. Data were collected from at least three independent experiments. Data were expressed as mean ± SEM and analyzed with the GraphPad Prism 5 software. All statistical tests were two-tailed. *p < 0.05, **p < 0.01, ***p < 0.001 were considered statistically significant.

## Results

### Morphology and distribution of AML-BM MSC subpopulations are impaired

To characterize the BM niche composition in AML, the study focused on two main subpopulations involved in AML tumorigenesis – osteocytes and adipocytes. Experiments were performed on primary non-induced and induced MSCs that were assessed for changes in their morphology and differentiation potential. H&E immunohistochemistry staining demonstrated fatty vacuolization and calcium deposits in induced adipocytes and osteocytes, respectively (Additional file 1: Fig. S1A, B). In both HD- and AML-MSCs, a significant and specific signal was demonstrated in induced adipogenic and osteogenic subpopulations, using flow cytometry analysis (Additional file 1: Fig. S1C).

### Levels of secreted FABP4 and osteocalcin biomarkers are reduced in AML-BMP

**Fig. 1:**
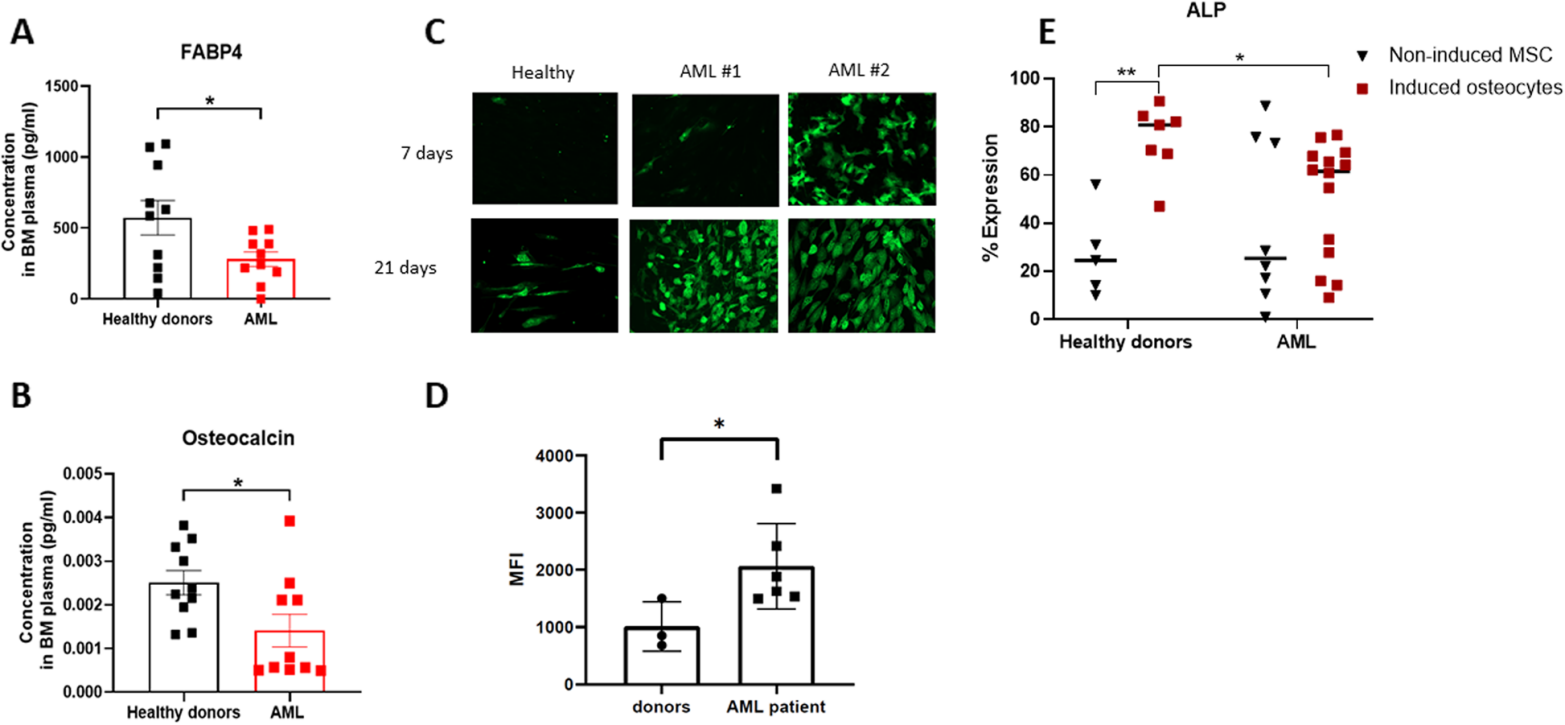
Differentiation potential of BM niche subpopulations. **A-B** BM plasma samples were collected from HDs (*n* = 10) and AML patients at diagnosis (*n* = 10). Adipocyte and osteocyte fractions were analyzed with the ELISA test for FABP4 and osteocalcin biomarkers, respectively. **C** Immunofluorescence staining of FABP4 in adipocytes derived from AML patients and HD at two time points (7 and 21 days) during the differentiation process. The scale bar is 10 µM. **D** Quantitative analysis of FABP4 expression levels, using FIJI software for fully differentiated (21 days) adipocytes derived from 6 AML patients and 3 HDs. MFI: median fluorescence intensity. **E** Protein expression of alkaline phosphatase (ALP) in induced osteocytes derived from 14 AML patients and 7 HDs, as evaluated using flow cytometry analysis. Non-induced MSCs derived from 8 AML patients and 5 HDs were used as control. Data are presented as mean ± SEM, with each patient sample presented as a dot or a unique symbol. Statistical analyses included: unpaired t test in A, D and E; Mann–Whitney U test in B. *P < 0.05 was considered statistically significant

The ELISA evaluation of the adipogenesis and osteogenesis status revealed significantly reduced levels of secreted FABP4 and osteocalcin biomarkers in the BMP of newly-diagnosed AML patients compared to HDs (FABP4: 572.8 ± 364.27 pg/ml and 279.7 ± 156.4 pg/ml, respectively; *p* = 0.039; osteocalcin: 0.00251 ± 0.000832 pg/ml and 0.00141 ± 0.001128 pg/ml, respectively; *p* = 0.03) (Fig. 1A, B). These data may reflect an impaired function of both adipocyte and osteocyte BM subpopulations in AML patients already at diagnosis.

### Differentiation capacity of MSCs into adipogenic and osteogenic lineages is impaired in AML

To determine whether the expanded MSC cultures possessed normal differentiation capacity towards adipogenic and osteogenic lineages in vitro, MSCs obtained from 14 AML patients at diagnosis and from 7 HDs were tested. The levels of FABP4 and ALP intracellular markers, measured by immunofluorescence staining and flow cytometry, respectively (Fig. 1C-E; Additional file 1: Fig. S1C), pointed to a different differentiation capacity of AML- and HD-MSCs.

Specifically, the findings of immunofluorescence staining using the anti-human FABP4 polyclonal antibody demonstrated a significant increase in the expression of FABP4 in AML-derived adipocytes compared to HD-derived adipocytes (2064 ± 33.06% and 1010 ± 35.0%, respectively; *p* = 0.03; Fig. 1C, D). The ALP expression levels in induced osteocytes were significantly lower in AML samples than in those of HDs (61.5 ± 22.7% and 80.8 ± 14.4%, respectively; *p* = 0.02; Fig. 1E).

The FABP4 gene expression, evaluated by qPCR, displayed no significant difference in either AML-induced versus HD-induced adipocytes or non-induced AML- versus HD-MSCs (Additional file 1: Fig. S2). Likewise, the PPARγ gene expression was non-significantly higher in AML- compared to HD-induced adipocytes (1.6 ± 0.5 and 1.0 ± 0.1, respectively; *p* = NS; data not shown). The obtained results may reflect an altered adipogenic signaling pathway in AML.

### Decreased AHNAK2 expression in AML induced adipocytes and osteocytes is related to the abnormal function of this gene

**Fig. 2:**
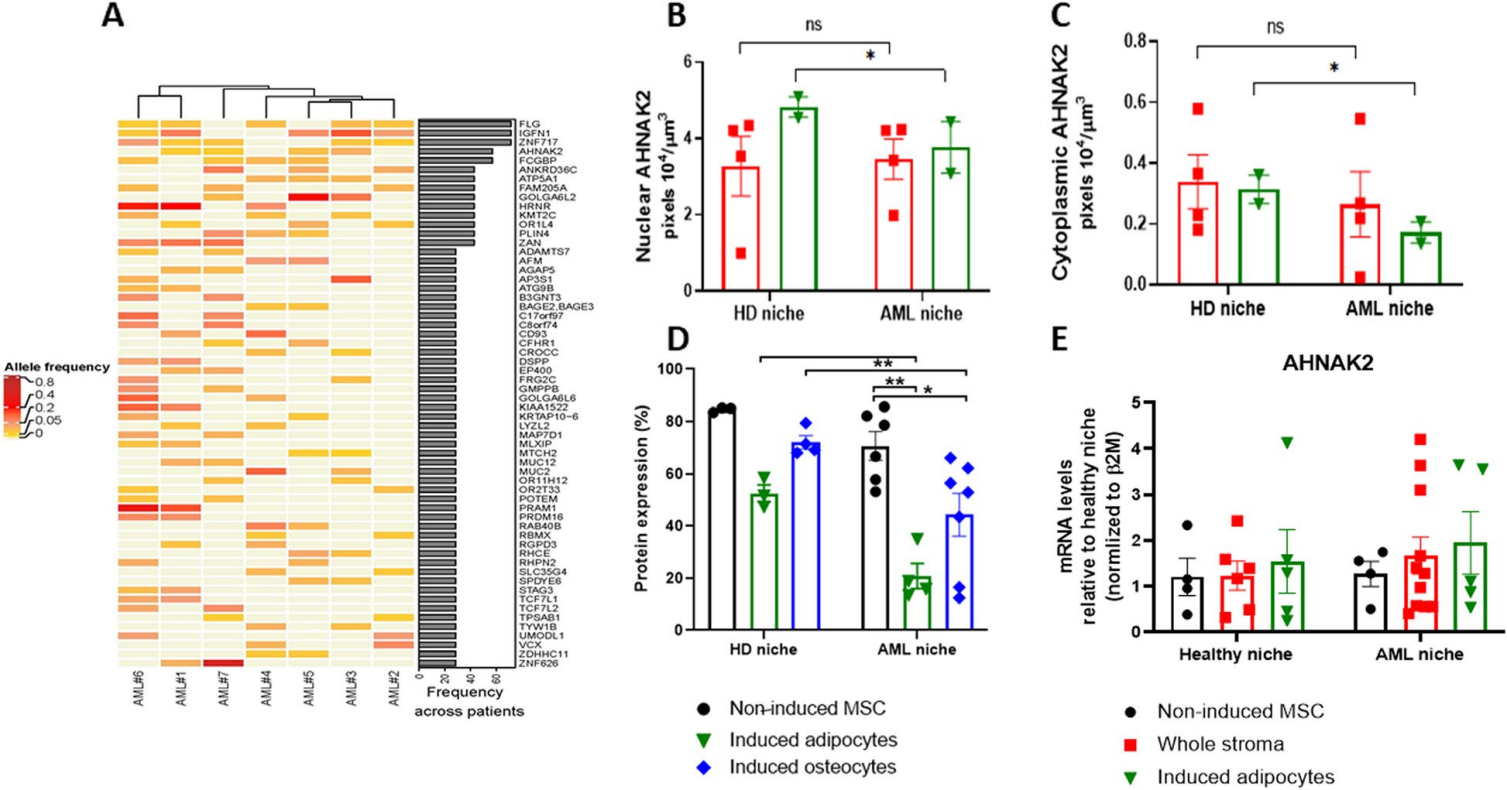
Molecular alterations in stroma cells of AML patients and functional analysis of AHNAK2 protein. **A** A heat-map illustrating DNA gene variations, identified using whole exome sequencing in samples derived from 7 AML patients. The colors represent allele frequencies of each single-nucleotide polymorphism (SNP). The right bar histogram indicates frequency across patients. **B**, **C** Nuclear and cytoplasmic quantitative analysis of AHNAK2 expression levels in MSCs and induced adipocytes, derived from 4 AML patients carrying an *AHNAK2* mutation and 4 HDs, using IMARIS software. **D** Flow cytometry analysis of AHNAK2 expression levels in MSCs derived from 6 AML patients and 3 HDs, as well as in induced adipocytes derived from 3 AML patients and 3 HDs and in induced osteocytes derived from 7 AML patients and 4 HDs. **E** Real-time PCR analysis of: MSCs derived from 4 AML patients and 4 HDs; whole stroma derived from 12 AML patients and 6 HDs; induced adipocytes derived from 6 AML patients and 5 HDs. *AHNAK2* was calibrated to *β2M* housekeeping gene. Data are presented as mean ± SEM

In stromal cells derived from 7 AML patients, somatic mutations were identified in 60/314 genes, using whole exome sequencing, with inter-patient differences demonstrated both in gene occurrence and allele frequency (Fig. 2A; Additional file 1: Fig. S3; Additional file 2: Table S4). To ensure a homogeneous composition of the stromal cells, the analysis was performed after 2–3 passages. Notably, 5/60 genes were present in more than 50% of patients, with the allele frequency ranging between 5%-60%. Among these 5 genes, *FLG* and *AHNAK2* were the only ones known to be associated with the BM niche, as they are involved in cell adhesion and calcium hemostasis, respectively.

To test if there is an abnormal translocation of the AHNAK2 protein between the nucleus and the cytoplasm, immunofluorescence staining was performed (Fig. 2B, C; Additional file 1: Fig. S3) in stromal cells of 4 HDs, 4 AML patients with mutated *AHNAK2* and in induced adipocyte cells derived from the above-mentioned 8 individuals (HD: 3270 ± 1580; AML: 3670 ± 1986 cells). There was no difference in the localization of the AHNAK2 protein between AML and HD stromal cells. The expression level of this protein was similar in the nucleus (3.41 ± 0.52 pixels 10^4^/μm^3^ and 3.22 ± 0.78 pixels 10^4^/μm^3^, respectively; *p* = 0.48), and the cytoplasm (0.26 ± 0.10 pixels 10^4^/μm^3^ and 0.33 ± 0.08 pixels 10^4^/μm^3^, respectively; *p* = 0.68) of the evaluated non-induced stromal cells. A significant decrease in the protein nuclear expression was detected in AML-induced adipocytes relative to those of HDs (3.76 ± 0.67 pixels 10^4^/μm^3^ and 4.83 ± 0.16 pixels 10^4^/μm^3^ respectively; *p* = 0.02; Fig. 2B). Likewise, significant reduction in the protein cytoplasmic expression was revealed in AML-induced adipocytes compared to their levels in HDs (0.17 ± 0.035 pixels 10^4^/μm^3^ and 0.3145 ± 0.02 pixels 10^4^/μm^3^, respectively; *p* = 0.01; Fig. 2C). This was further verified by flow cytometry analysis, demonstrating no change in the protein level in non-induced MSCs of both HD and AML patients (HD: 84.5 ± 0.65% vs AML: 70.7 ± 6.03%; *p* = 0.12; Fig. 2D), while a significant reduction was observed in induced adipocytes and osteocytes in AML patients relative to HD (HD: 52 ± 3.95% vs AML: 20.8 ± 4.97%; *p* = 0.004; HD: 72 ± 2.97% vs AML: 44.3 ± 8.83%; *p* = 0.03). MSCs, whole stroma, induced adipocytes and osteocytes demonstrated no significant differences in the AHNAK2 mRNA expression between AML patients and HDs (Fig. 2E). This suggests that the decrease in the AHNAK2 expression in AML induced adipocytes and osteocytes is related to the protein function rather than to its production.

### AML-MSC subpopulations support the survival of leukemic blasts/progenitors and their clonogenic potential

**Fig. 3:**
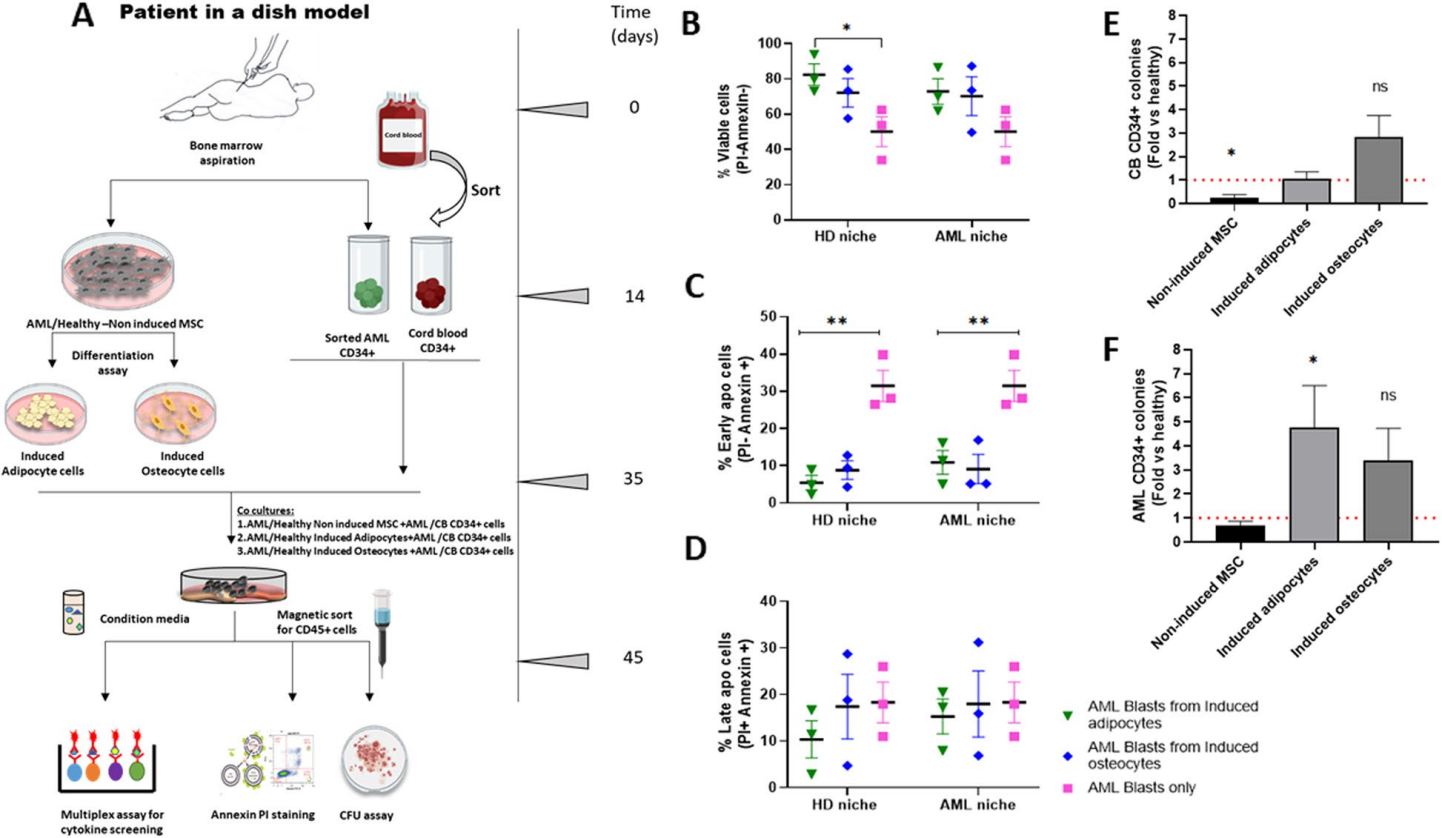
Effects of AML stromal fractions on hematopoiesis and leukemogenesis, evaluated using the “patient-in-a-dish” system. **A** A “patient-in-a-dish” ex-vivo system mimicking the AML-ME and allowing to investigate a crosstalk between BM-derived adipocyte and/or osteocyte subpopulations and leukemic cells, was generated. The schema presents all the steps of the experimental setup. **B-D** Cell viability analyses of apoptotic levels of blast cells upon their co-culture. Data are presented as mean ± SEM, with each patient sample presented as a dot (AML patients: *n* = 3; HDs: *n* = 3). The unpaired t test was used for statistical analysis. *P < 0.05 and **P < 0.01 were considered statistically significant. **E**, **F** Colony forming unit assay was used to distinguish between normal and leukemic hematopoiesis. The number of colonies in samples derived from AML patients was normalized to that found in samples derived from HDs. The red dotted line represents HD levels

To explore whether AML aberrant adipocyte and/or osteocyte subpopulations provide preferential support to leukemic cell survival and proliferation, we created a”patient-in-a-dish” system (Fig. 3A). The viability of AML blasts co-cultured with induced adipocytes/osteocytes derived from 3 HDs and 3 AML patients (HD adipocytes: 82 ± 6%, AML blasts: 50 ± 8%; *p* = 0.02; HD osteocytes: 72 ± 8%, AML blasts: 50 ± 8%; *p* = 0.06) was greater than that of AML blasts cultured alone (adipocytes: 72 ± 7%, blasts: 50 ± 8%, *p* = 0.05; osteocytes: 70 ± 10%, blasts: 50 ± 8%; *p* = 0.11; Fig. 3B). Early apoptosis assays demonstrated a higher apoptosis level in AML blasts cultured alone compared to the above-mentioned co-cultures (AML blasts: 31 ± 4% versus HD adipocytes: 5 ± 1%, *p* = 0.006; AML blasts: 31 ± 4% versus HD osteocytes: 8 ± 2%, *p* = 0.007; AML blasts: 31 ± 4% versus AML adipocytes: 10 ± 3%; *p* = 0.01; AML blasts: 31 ± 4% versus AML osteocytes: 9 ± 3%; *p* = 0.008; Fig. 3C). No differences in early apoptosis between the evaluated co-cultures were observed. These findings may imply that both normal and AML stromal cells provide supportive ME for AML cell survival. A similar trend was found in late apoptosis measurements (HD adipocytes: 10 ± 4%, HD osteocytes: 17 ± 6%, AML adipocytes: 15 ± 3%, AML osteocytes: 17 ± 7%, AML blast: 18 ± 4%; Fig. 3D).

The MSC potential to support proliferation of CD34 + AML blasts was assessed using CFU assays in induced and non-induced AML- and HD-MSCs. Significantly diminished formation of CFU-E (erythrocyte colonies) compared to CB CD34 + CFU was demonstrated in AML samples (Fig. 3E, F; Additional file 1: Fig. S4A, B). Upon normalization to the HD-MSCs values, the following increase in the number of colonies was observed: 0.24 ± 0.14-fold (*p* = 0.02), 1.05 ± 0.29-fold (*p* = 0.1) 2.84 ± 0.89-fold (*p* = 0.31) for non-induced AML-MSCs, induced AML adipocytes and induced AML osteocytes, respectively (Fig. 3E). The corresponding values for an increase in the AML-derived colony growth equated to: 0.67 ± 0.18-fold (*p* = 0.14), 4.77 ± 1.73-fold (*p* = 0.02) and 3.37 ± 1.35-fold (*p* = 0.1), respectively (Fig. 3F).

Overall, unlike HD-MSCs, non-induced AML-MSCs decreased the proliferative potential of normal hematopoiesis (CB cells), while induced adipocytes/osteocytes did not significantly alter this property. Conversely, the leukemogenic potential (AML-CFU) was significantly more pronounced in AML-induced adipocytes relative to those of HDs, whereas AML-induced osteocytes did not differ in their support of hematopoiesis.

### The cytokine flux is altered in co-cultures of leukemic cells and BM subpopulations in AML

The investigation of potential communication pathways between AML cells and different BM niche subpopulations, using the Multiplex assay, showed the following changes in the cytokine flux normalized to the levels measured in corresponding cell fractions derived from HDs: a significant reduction of IFNγ (0.7-fold, *p* = 0.0002) and IL-8 (0.7-fold, *p* = 0.012) levels along with a significant increase of SDF1-alpha (1.34-fold, *p* = 0.04) levels in AML cells co-cultured with non-induced AML-MSCs compared to non-induced HD-MSCs. Furthermore, a significant elevation in IL-10 (1.34-fold, *p* = 0.04) levels was demonstrated in co-cultures containing AML-induced adipocytes relative to HD-induced adipocytes. As to the osteocyte subpopulation, SDF1-alpha levels were significantly reduced (0.63-fold, *p* = 0.004) in co-cultures containing AML- compared to HD-induced osteocytes. These findings could reflect pronounced impairment in the crosstalk between leukemic cells and certain BM subpopulations in AML. Notably, levels of other evaluated cytokines were unaffected (Additional file 1: Fig. S5).

### The BM adipocyte subpopulation provides preferential support to AML cell survival and proliferation

**Fig. 4:**
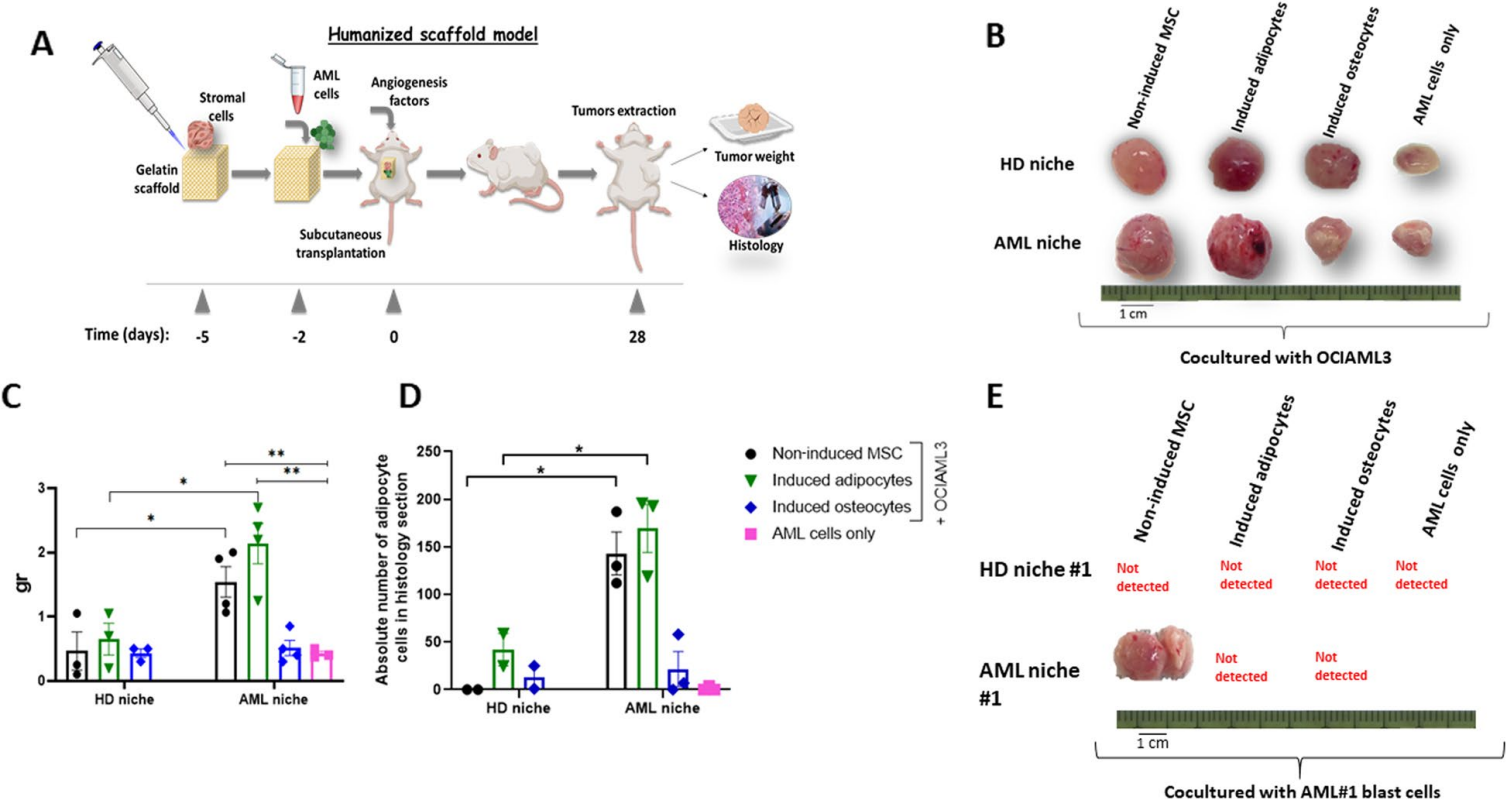
Interactions between AML cells and different BM cell subpopulations investigated using the 3D humanized scaffold xenograft model. **A** Schematic presentation of the experimental setup. **B** Representative images of the tumor mass formed on the scaffolds in the presence of different mesenchymal cell fractions. **C** Plots representing the dry tissue weights of the tumors formed on the scaffolds in the presence of different mesenchymal cell fractions. One paired Mann Whitney was used for statistical analysis. *P < 0.05 was considered statistically significant. **D** Plots representing the absolute number of adipocyte cells in the sections demonstrated in Fig. S7A, assessed using the image j tool. Data are presented as mean ± SEM. The unpaired t test was used for statistical analysis. *P < 0.05 was considered statistically significant. **E** Interactions between AML cells and different BM cell subpopulations investigated using the fully humanized 3D scaffolds. Representative images of the tumor mass formed on the scaffolds in the presence of different mesenchymal cell fractions and AML blasts derived from 4 AML patients

A modified 3D humanized scaffold system, allowing to monitor the movement of live human hematopoietic leukemic cells to the humanized BM-ME and the formation of the vasculature and tumor tissue, was employed to explore in-vivo the study findings delineated above (Fig. 4A).

In samples derived from 4 AML patients, a significantly larger tumor mass was observed in the scaffolds coated with AML-non-induced MSCs and AML-induced adipocytes relative to the scaffolds coated with AML-induced osteocytes and all the control combinations with HD cell subpopulations (Fig. 4B). Strong CD45 staining was detected in the tumor tissue, confirming human cell engraftment and survival of the OCIAML3 cell-line in humanized scaffolds (Additional file 1: Fig. S6A). The anti-human FABP4 protein expression confirmed the presence of human adipocytes in tumors (Additional file 1: Fig. S6B).

Significantly greater tumor mass weight was documented in AML non-induced and induced adipocytes relative to the corresponding HD-fractions (1.54 ± 0.23 g and 2.13 ± 0.31 g versus 0.46 ± 0.29 g and 0.65 ± 0.24 g, respectively; *p* = 0.02). Importantly, the osteocyte-induced stroma provided a similar support to tumor growth, irrespective of the stroma origin (0.51 ± 0.11 g versus 0.43 ± 0.06 g, respectively; Fig. 4C). Tumors formed on the scaffolds in the presence of non-induced AML-MSCs (143 ± 22 cells) and AML-induced adipocytes (169 ± 14 cells) contained a significantly higher number of adipocytes than HD-MSCs (0 cells) and HD-induced adipocytes (42 ± 17 cells; *p* = 0.01 and *p* = 0.03, respectively; Fig. 4D; Additional file 1: Fig. S7A). No adipocytes were detected in the scaffolds coated with induced osteocytes or AML cells alone.

The ability of adipocytes and osteocytes to support leukemia propagation was further investigated in the fully humanized scaffold system, specifically designed in the present study to conduct experiments, exclusively using primary MSCs and leukemic cells. Leukemic engraftment was detected only in the scaffolds seeded with non-induced AML-MSCs derived from AML patients (*n* = 4; Fig. 4E). Importantly, while AML-MSCs had been originally seeded on the scaffolds, only adipocytes emerged as the dominant component of the ME in the tumors extracted from the mice (Additional file 1: Fig. S7A, B). These findings strongly support the results obtained in the experiments where significantly larger tumor masses were observed in the scaffolds coated with AML-non-induced MSCs and AML-induced adipocytes (Fig. 4B). These data collectively point to preferential spontaneous differentiation of AML-MSCs into the adipocytes, that, in their turn, provided support to tumor progression.

## Discussion

Recent evidence demonstrates an essential role of the BM-ME in protecting tumor cells in AML [27]. Specifically, BM-MSCs, mainly differentiating into adipocyte, endothelial and/or osteoblast subpopulations, are known to support the expansion of the leukemia cell population and prevent its apoptosis [9, 15, 28, 29]. The reports evaluating late AML relapse post-allogeneic stem cell transplantation [30, 31] suggest that the BM niche may be a potential disease source. It remains unclear whether the primary alterations leading to leukemogenesis lie within the BM niche itself or result from its interaction with leukemia-initiating cells. Both processes can interact to generate an abnormal milieu supporting AML initiation/propagation.

There is a controversy regarding the differentiation potential of adipocytes and osteocytes in AML-mesenchymal cells that could affect normal hematopoiesis and lead to preferential support of leukemic HSPCs. While some studies point to an increased tendency to adipogenesis [13, 14], others demonstrate reduced adipogenic differentiation in AML-mesenchymal cells [16, 17, 32]. As to the osteocyte subpopulation, Battula et al. [16] have demonstrated preferential osteogenic differentiation in mesenchymal stromal cells in AML, whereas Geyh et al. [33] have found a reduced osteogenic differentiation potential along with a decreased expression of osteocalcin and osterix.

Results of the present study, obtained using morphological, ELISA, FACS and qPCR analyses, demonstrate differences between AML- and HD-derived MSCs in their capacity to differentiate into adipogenic and osteogenic lineages. Indeed, the impaired differentiation potential was found both in AML-induced adipocytes, as verified by significantly elevated FABP4 protein expression, and osteocytes, as confirmed by decreased osteocalcin levels. The fact that these aberrations have not been observed in non-induced MSCs points to disease-associated abnormalities in the differentiation pathway, which could lead to disruption of the balanced structure of various niches within the BM, eventually causing a change in their interaction with hematopoietic/leukemic cells.

Adipocytes are reported to exert anti-apoptotic effects on AML cells [34], with their presence resulting in increased fatty acid β-oxidation along with upregulation of PPARγ, CD36 and BCL2 proteins. Indeed, the current study has revealed elevated *PPAR*γ gene expression levels in AML- relative to HD-induced adipocytes. This may serve as a piece of evidence of an impaired adipogenic pathway, where the transcriptional factor PPARγ, preceding the FABP4 generation, accumulates [35]. Additionally, the AML-adipocyte niche is demonstrated to support leukemic clonogenicity.

Recent studies suggest that genetic alterations in the BM niche cells may cause myeloid leukemia in mouse models [29]. While these findings in mice offer direct evidence for mutated niche-induced leukemogenesis, emerging reports of donor cell leukemia in humans (1–5% of all post-transplant leukemia relapses), also infer a role of oncogenic ME in driving secondary malignancy [30, 36, 37]. It is suggested that genetic aberrations in the niche components could be a key event in AML initiation and progression. However, little is known about the genetic profile of specific mutations involved in these processes. In the current study, impaired genetic and functional properties of adipocytes and osteocytes have been found in AML patients already at diagnosis. Whole exome sequencing has identified 60 somatic mutations in AML stromal cells, with a mutated *AHNAK2* found among the five most frequently mutated genes (> 40% of patients). Of these genes, only *AHNAK2* has been previously reported to be directly related to tumor ME [22, 38–41] and has thus been chosen in this study for further investigation. In our experiments, *AHNAK2* mutations have been located in the conserved region of the gene and therefore might be involved in the 3D structure of the protein. The expression of the AHNAK2 protein appears to be decreased in AML-induced adipocytes and osteocytes, which has not been observed in non-induced MSCs. Whether this decline is related to incomplete and abnormal differentiation of these subpopulations or it is a possible factor affecting MSC stemness and differentiation, is yet to be determined.

The AHNAK2 protein is supposed to participate in calcium signaling and cytoarchitecture [22] and the BM niche-calcium homeostasis is suggested to be crucial during leukemia initiation [23]. Moreover, this protein is reported to mediate adipocyte differentiation, implying that AHNAK2 regulates adipogenesis by affecting the smad1-dependent PPARγ expression [21]. Our finding of reduced AHNAK2 protein expression solely in AML-induced adipocytes/osteocytes suggests potential involvement of this protein in impaired MSC differentiation toward adipocyte and osteocyte lineages in the AML-BM niche. Overall, the *AHNAK2* gene expression and protein levels could contribute to the aberrant molecular signature of the AML-BM niche components.

The involvement of signaling mediators, such as cytokines (e.g., IFNγ, IL-10, IL-8) and chemokines (e.g., SDF1-alpha), in the crosstalk between HSPCs and the BM niche is well-established [42–48]. In the present study, levels of these cytokines, normalized to those measured in corresponding HD-derived cell fractions, are found to be significantly elevated in AML-induced adipocytes/osteocytes relative to non-induced MSCs, which might point to suppressed hematopoiesis. Furthermore, an interaction of the MSC-produced chemokine SDF1-alpha with its receptor CXCR4 is known to be essential for HSPC maintenance, proliferation and homing to the BM [49]. Thus, significantly reduced SDF1-alpha levels in the co-cultures containing AML-induced osteocytes, observed in our study, could negatively affect this interaction. The abnormalities in the balance between BM subpopulations in AML patients, unraveled in the current study, and their contribution to the expansion of the AML cell population may be attributed to an impaired crosstalk mediated by cytokines and chemokines, secreted by these cells. Such abnormal crosstalk may contribute to creating the BM-ME supportive of leukemia development.

Using the 3D humanized scaffold xenograft model, originally proposed by the Bonnet group [25] to recapitulate physiological interactions between AML cells and different BM cell subpopulations, our study has demonstrated the inhibition of normal CD34 + cell expansion in AML [50]. Furthermore, mesenchymal stromal cells derived from the AML-BM are reported to enhance leukemia cell proliferation [32]. In the present study, the 3D scaffold model has been employed to explore in-vivo the involvement of specific BM niche components in AML development. Similar to other studies, our experiments have shown an increased capacity of AML-MSCs to maintain leukemogenesis compared to HD-MSCs. Moreover, to the best of our knowledge, the current study is the first to document in-vivo, using a fully humanized scaffold system, exclusively utilizing patient-derived cells, a robust support provided by the AML-induced adipogenic subpopulation to leukemia cell survival and tumor growth relative to the AML/HD osteogenic and non-induced MSC niches. Actually, tumor histology dissection revealed that upon transplantation of non-induced AML-MSCs to mice, these cells spontaneously differentiated into the adipogenic fraction that, in its turn, preferentially supported the leukemogenesis.

This study has several limitations. All the experiments have been conducted using primary cells derived from either AML patients at diagnosis or HDs. While this is an advantage allowing to design an accurate experimental setup, it is also associated with certain limitations due to high inter-patient variability and a restricted number of cells that could be used in each experiment. To overcome these shortcomings, a sufficient number of human samples has been obtained to draw definitive conclusions. The presented approach for the investigation of regulatory mechanisms of adipogenesis/osteogenesis in AML requires additional steps that are currently under development at our laboratory.

## Conclusions

Overall, the current study has demonstrated a direct contribution of abnormal functional, genetic and molecular properties of AML BM-derived adipocytes and osteocytes to the leukemogenesis-supportive ME, which is particularly associated with the involvement of the AML adipocyte subpopulation. AML-MSCs are found to preferentially and spontaneously differentiate into the adipocyte cells, thus expanding their fraction and making them the dominant subpopulation in the AML BM. These adipocytes appear to provide significant support to tumor progression. These findings may serve as a platform for a novel therapeutic strategy in AML management, targeting disease-originating cell subpopulations in the BM rather than leukemic cells in the peripheral blood and thus preventing disease relapse.

### Supplementary Information

**Additional file 1: Fig. S1.** Analysis of differentiation potential of primary MSCs toward adipogenic and osteogenic lineages. **Fig. S2.** Evaluation of *FABP4 *and *PPARγ* expression levels on patient and HD cells using qPCR analyses. **Fig. S3.** Immunofluorescence staining used to determine AHNAK2 protein levels in AML and HD stromal cell cultures. **Fig. S4.** Survival and clonogenic potential of AML- and HD-MSC derived subpopulations. **Fig. S5.** Levels of secreted cytokines evaluated using the “patient-in-a-dish” system. **Fig. S6.** Confirmation of cell engraftment. **Fig. S7.** Representative images of H&E immunohistochemistry staining.

**Additional file 2: Table S1.** Diagnosis and characteristics of AML study cohort. **Table S2.** Diagnosis and characteristics of HD study cohort. **Table S3.** Antibodies used in the study. **Table S4.** Genetic alterations. **Table S5.** Primers used for sequencing.

## Data Availability

The data that support the findings of this study are available from the corresponding author upon reasonable request.

## Abbreviations

ALP: Anti-human alkaline phosphatase
AML: Acute myeloid leukemia
ANG1: Angiopoietin 1
BM: Bone marrow
BM-MSCs: BM mesenchymal stem cells
BMP: Bone marrow plasma
CB: Cord blood
CFU: Colony forming unit
FABP4: Fatty-acid-binding protein 4
FBS: Fetal bovine serum
FITC: Fluorescein isothiocyanate
GM-CSF: Granulocyte–macrophage colony-stimulating factor
H&E: Hematoxylin–eosin
HD: Healthy donors
HSPCs: Hematopoietic stem and progenitor cells
IDO: Indoleamine 2,3-dioxygenase
IFN-ɣ: Interferon ɣ
IL-6: Interleukin 6
LAIP: Leukemia-associated immunophenotype
MCP-1: Monocyte chemoattractant protein-1
ME: Microenvironment
MEM-α: Minimum essential medium-α
MNC: Mononuclear cells
MSCs: Mesenchymal stem cells
OPN: Osteopontin
P: Passage
PI: Propidium iodide
RT: Room temperature
RT-qPCR: Real-time quantitative polymerase chain reaction
SCF: Stem cell factor
TNF-α: Tumor necrosis factor α
TPO: Thyroid peroxidase
