# Supplementary material for: Abnormal adipogenic signaling in the bone marrow mesenchymal stem cells contributes to supportive microenvironment for leukemia development
Source: Cell Commun Signal. 2023 Oct 10;21:277. doi: 10.1186/s12964-023-01231-z (PMC10563260; doi:10.1186/s12964-023-01231-z)
Supplement: Supplementary file 2 — Additional file 1: Fig. S1. Analysis of differentiation potential of primary MSCs toward adipogenic and osteogenic lineages. Fig. S2. Evaluation of FABP4 and PPARγ expression levels on patient and HD cells using qPCR analyses. Fig. S3. Immunofluorescence staining used to determine AHNAK2 protein levels in AML and HD stromal cell cultures. Fig. S4. Survival and clonogenic potential of AML- and HD-MSC derived subpopulations. Fig. S5. Levels of secreted cytokines evaluated using the “patient-in-a-dish” system. Fig. S6. Confirmation of cell engraftment. Fig. S7. Representative images of H&E immunohistochemistry staining. [file 12964_2023_1231_MOESM1_ESM.doc]

**Supplementary Information**

**Additional file: Fig S1-S7**

**
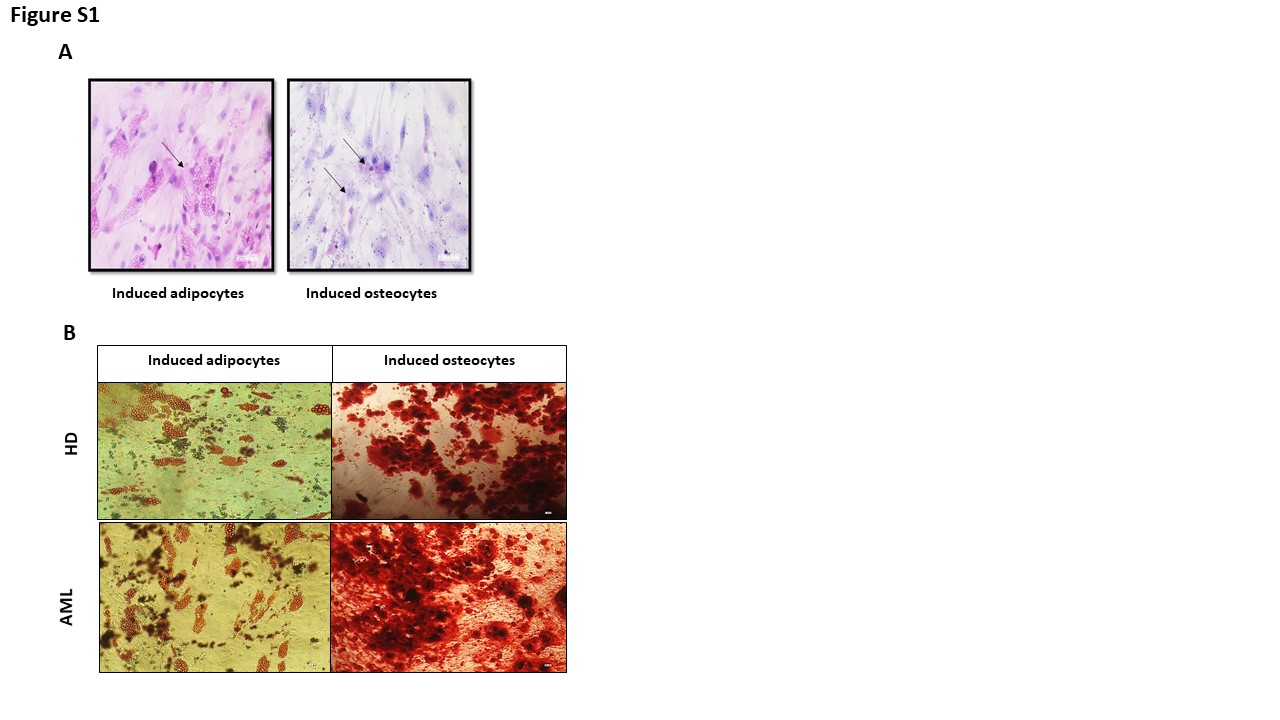

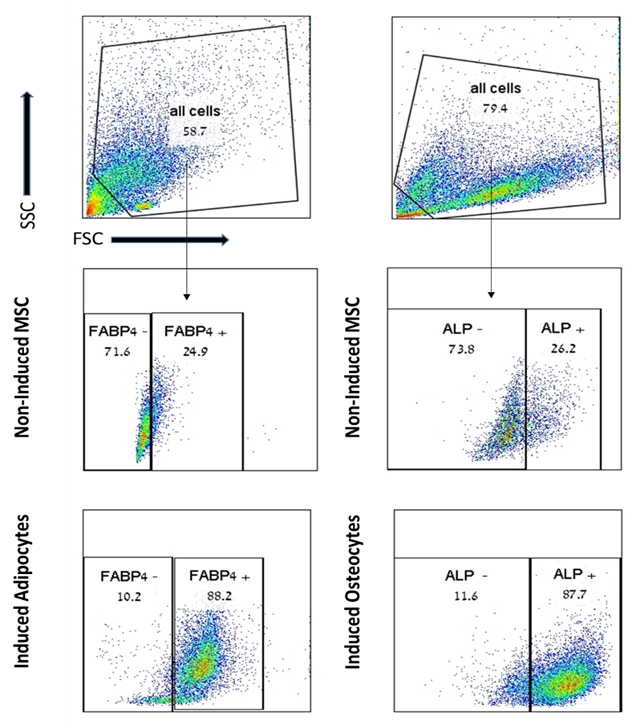
**

**C**

**Fig S1 Analysis of differentiation potential of primary MSCs toward adipogenic and osteogenic lineages**

**A** H&E immunohistochemistry staining of human MSC-derived adipocytes and osteocytes.

**B** Representative images of adipocyte (after 21 days) and osteocyte (after 14 days) differentiation in MSCs derived from HDs (upper panels) and AML patients (lower panels), using Alizarin Red and Oil Red, respectively. Scale bar = 100 μm

**C** Flow chart of flow cytometry analysis identifying differentiated adipocytes (FABP4+) and osteocytes (ALP+). Propidium iodide (PI) was used to distinguish between dead and live cells.

**
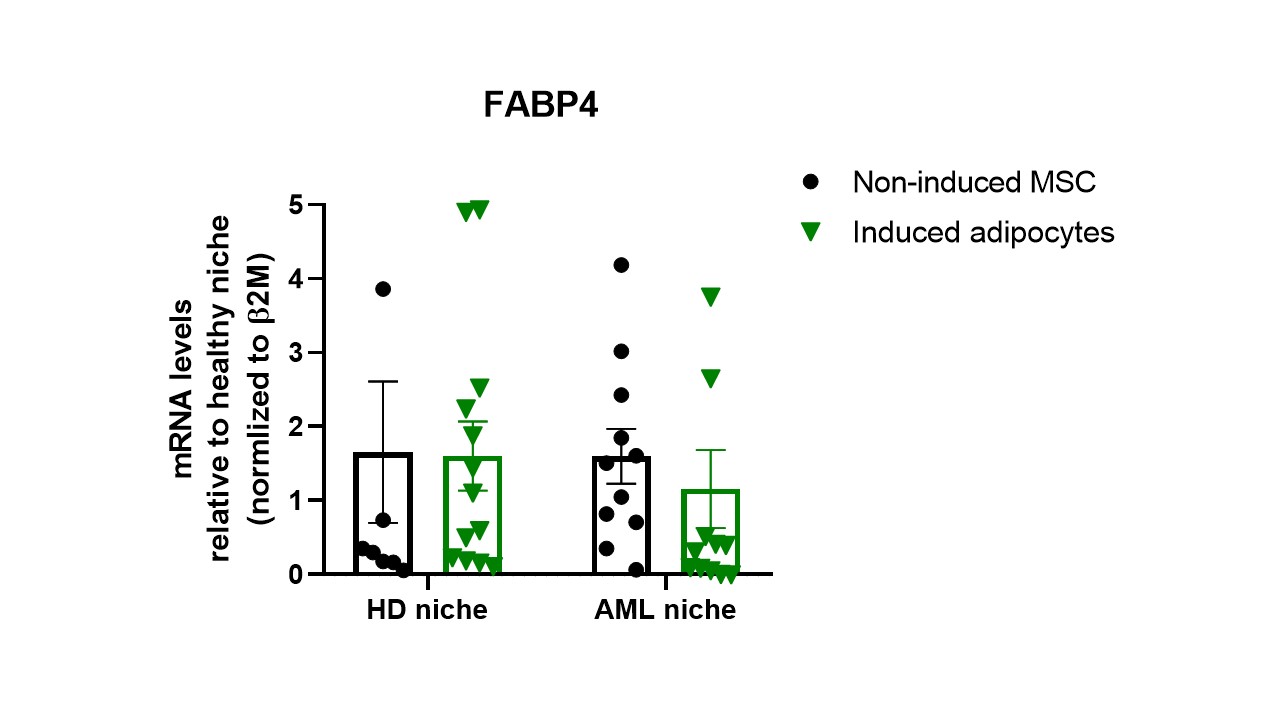
**

**Fig S2 Evaluation of *FABP4* and *PPARγ* expression levels on patient and HD cells using qPCR analyses**

Levels of *FABP4* expression in non-induced MSCs (n=3) and induced adipocytes derived from AML patients (n=4) and HDs (n=5).

The results were normalized to *β2M* housekeeping gene and the calculations were presented as a fold change relative to HD average values. Data are presented as mean ± SEM. The one-paired Mann-Whitney U test was used for statistical analysis. *P <0.05; **P <0.01 and ***P <0.001 were considered statistically significant.


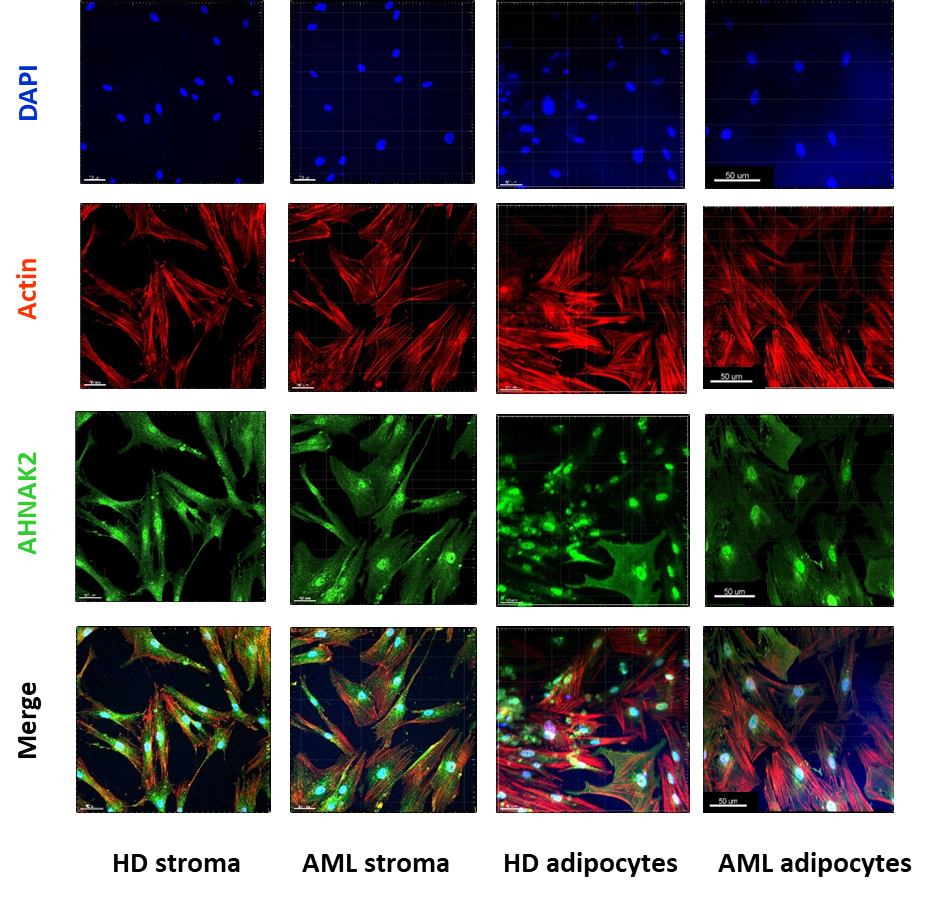

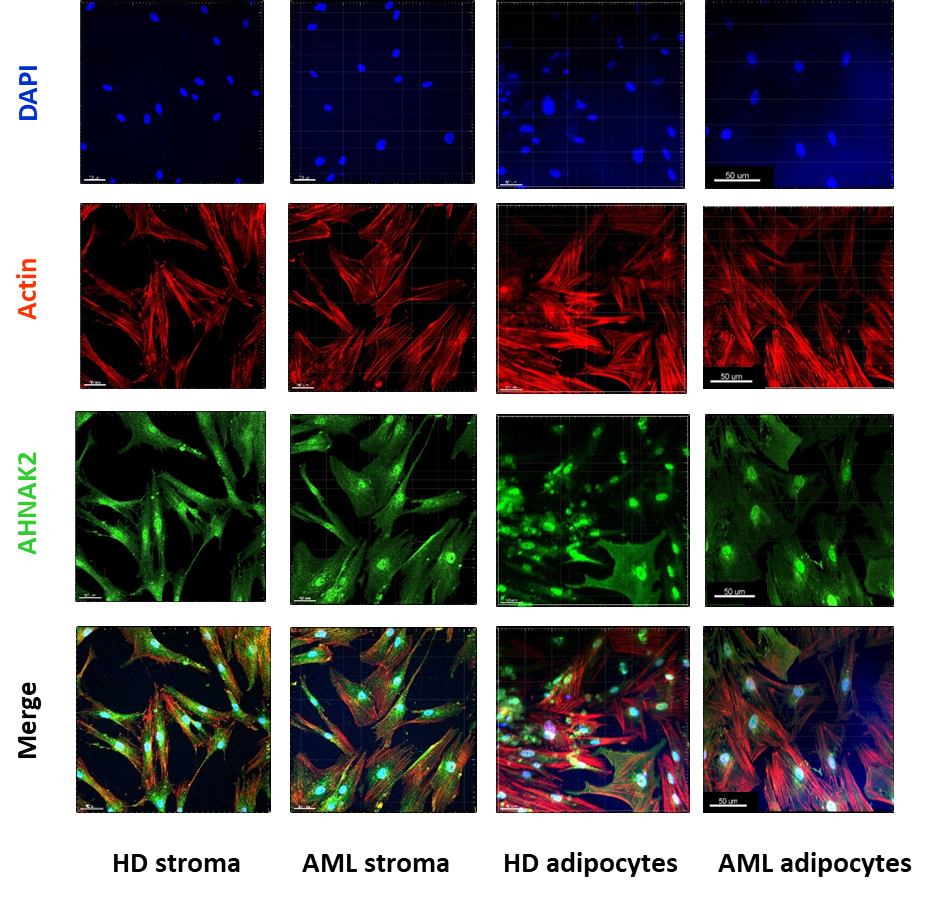


**Fig S3 Immunofluorescence staining used to determine AHNAK2 protein levels in AML and HD stromal cell cultures**

Scale bar =20 µm.


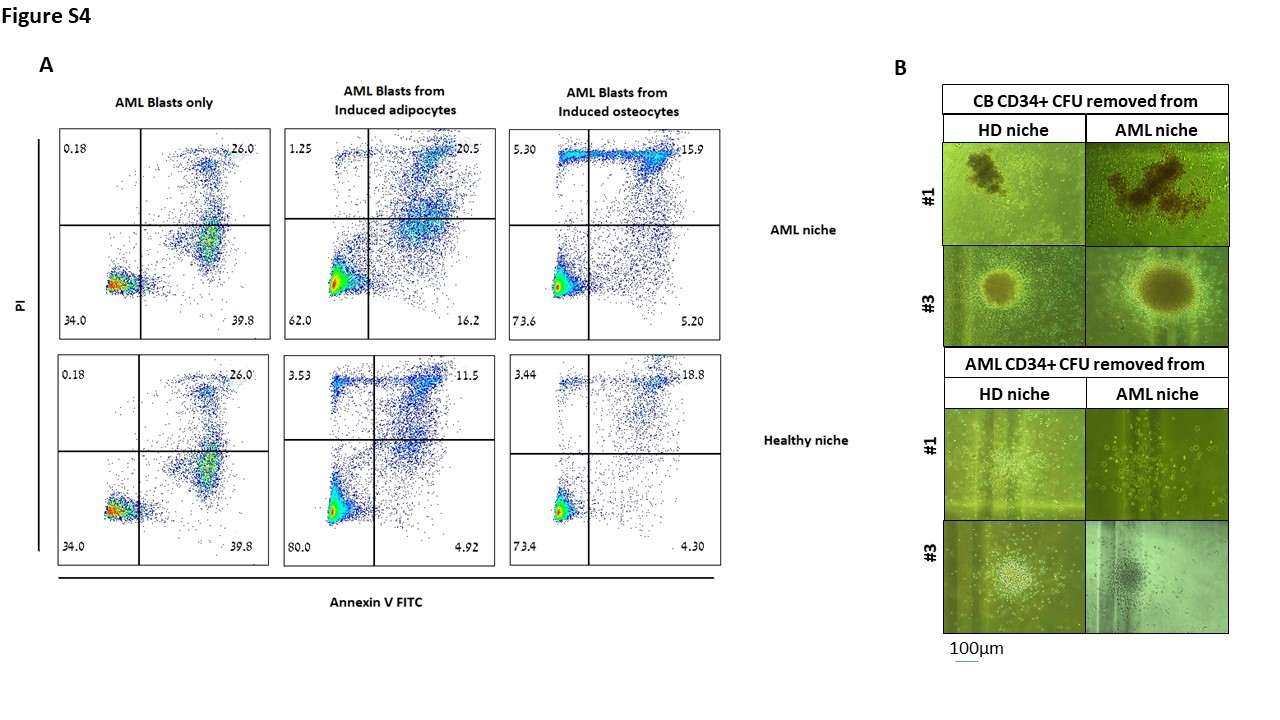


**Fig S4 Survival and clonogenic potential of AML- and HD-MSC derived subpopulations**

**A** Representative FACS plots for Annexin V/PI assay of AML blast cells co-cultured with AML-derived non-induced MSCs, induced adipocytes and induced osteocytes.

**B** Representative images of colonies formed in the colony forming unit assay.


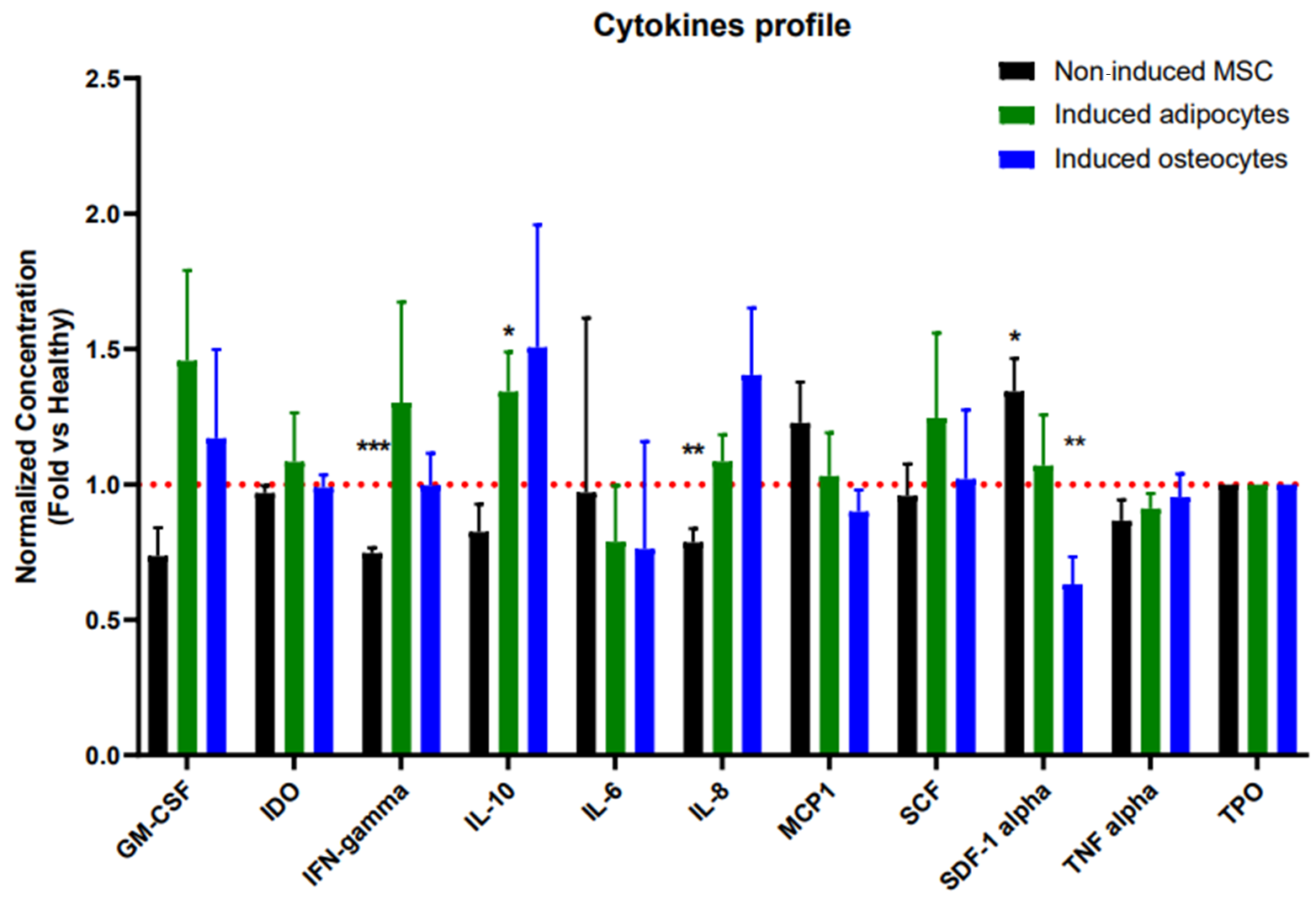


**Fig S5 Levels of secreted cytokines evaluated using the “patient-in-a-dish” system**

Cytokine secretion profile was quantified with a ProcartaPlex multiplex immunoassay kit (eBioscience). Levels of 11 evaluable cytokines were assessed in samples derived from AML patients (n=6) and HDs (n=6). Data are presented as mean ± SEM. The red dotted line represents HD levels. The unpaired t test was used for statistical analysis. *P <0.05; **P <0.001 and ***P <0.0001 were considered statistically significant.


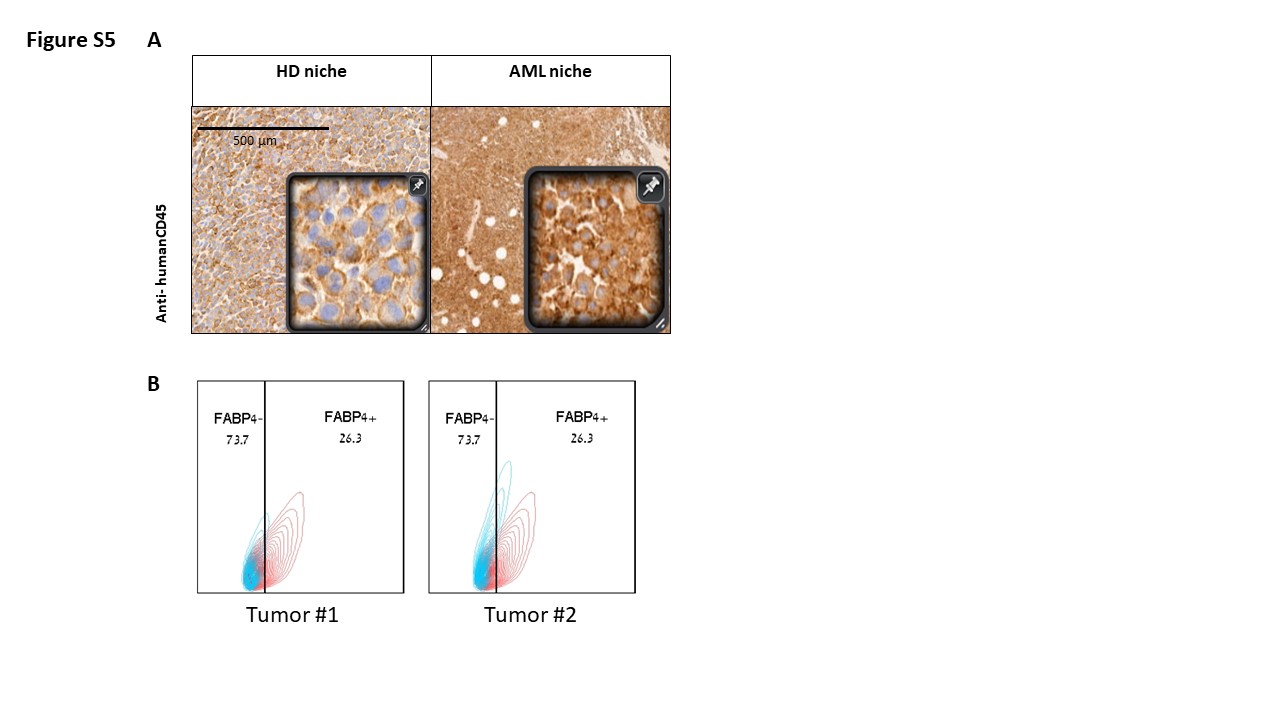


**Fig S6 Confirmation of cell engraftment**

**A** Images of two-photon confocal microscopy presenting CD45 staining results confirming engraftment.

**B** FACS plots presenting FABP4 expression levels in two tumor samples extracted from in-vivo humanized scaffolds.

**A**

**B**


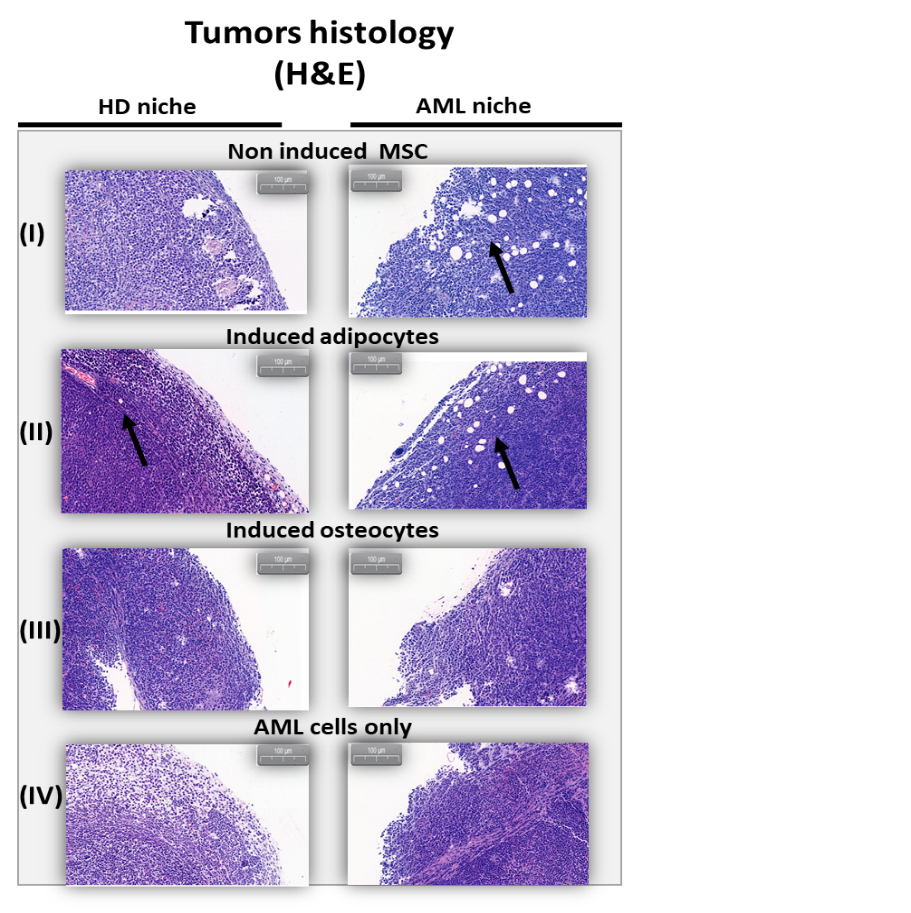


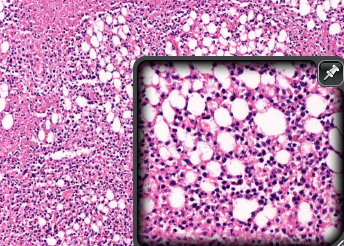


500 µm

**AML- non-induced MSC**

**Tumors histology**

**Fig S7 Representative images of H&E immunohistochemistry staining**

**A** Performed on the tumors formed on the scaffolds

**B** Performed on the tumors formed in-vivo on the scaffolds.
