# Supplementary material for: Abnormal adipogenic signaling in the bone marrow mesenchymal stem cells contributes to supportive microenvironment for leukemia development
Source: Cell Commun Signal. 2023 Oct 10;21:277. doi: 10.1186/s12964-023-01231-z (PMC10563260; doi:10.1186/s12964-023-01231-z)
Supplement: Supplementary file 3 — Additional file 2: Table S1. Diagnosis and characteristics of AML study cohort. Table S2. Diagnosis and characteristics of HD study cohort. Table S3. Antibodies used in the study. Table S4. Genetic alterations. Table S5. Primers used for sequencing. [file 12964_2023_1231_MOESM2_ESM.doc]

**Supplementary Information**

**Additional file: Tables S1-S5**

**Table S1 Diagnosis and characteristics of AML study cohort**

| **Lab. code** | **FAB subtype** | **Cytogenetics** | **Karyotype** | **%Blasts** | **ELN score** | **Sex** | **Age** |
| --- | --- | --- | --- | --- | --- | --- | --- |
| 519 | M1/M2 | Normal | *NPM1- FLT3-* | 37 | Intermediate | M | 40 |
| 490 | M5 | Normal | *NPM1- FLT3-* | 65 | Intermediate | M | 64 |
| 499 | M1/M2 | inv(16) | *NPM1- FLT3- (kit exom 8 +)* | 65 | Favorable | F | 56 |
| 489 | M4 | Normal | *NPM1- FLT3-* | 90 | Intermediate | M | 75 |
| 541 | M1 | Normal | *NPM1+ FLT3-* | 75 | Favorable | F | 40 |
| 506 | M1/M2 | Normal | *NPM1- FLT3-* | 61 | Intermediate | F | 40 |
| 493 | M4 | Normal | *NPM1+ FLT3-* | 65 | Favorable | M | 56 |
| 573 | M4 | Normal | *NPM1- FLT3-* | 22 | Intermediate | M | 55 |
| 536 | M1/M2 | NA | *NPM1- FLT3-* | 20 | NA | F | 38 |
| 516 | M1/M2 | Normal | *NPM1+ FLT3+* | 34 | Intermediate | M | 54 |
| 553 | NA | Normal | *NPM1+ FLT3+* | 56 | Intermediate | F | 76 |
| 556 | NA | t(8:21) | *NPM1- FLT3-* | 45 | Favorable | F | 40 |
| 566 | NA | del(20), +8 | *NPM1+ FLT3+* | 55 | Intermediate | F | 74 |
| 460 | M1/M2 | Normal | *NPM1- FLT3+* | 45 | Adverse | F | 26 |
| 465 | B-phenotype | t(9:22) | *NPM1- FLT3-* | 48 | Adverse | M | 22 |
| 545 | M4 | Normal | *NPM1- FLT ITD- FLT3 TKD+* | 75 | Intermediate | M | 71 |
| 447 | M5 | Normal | *NPM1- FLT3-* | 50 | Intermediate | M | 66 |
| 440 | M4/M5 | Normal | *NPM1+ FLT3+* | 75 | Intermediate | M | 53 |
| 525 | M5 | Normal | *NPM1+ FLT3+* | 60 | Intermediate | M | 62 |
| 547 | NA | Normal | *NPM1- FLT3-* | 27 | Intermediate | F | 74 |
| 572 | M4 | inv(16) | *NPM1- FLT3-* | 50 | Favorable | F | 44 |
| 502 | M4 | Normal | *NPM1+ FLT3+* | 65 | Intermediate | M |  |
| 577 | NA | Normal | *NPM1- FLT3-* | 68 | Intermediate | F | 19 |
| 581 | M1/M2 | t(8,21), del(9) | *NPM1- FLT3-* | 50 | Favorable | M | 50 |
| 554 | M5 | t(10,11), del(9) | *NPM1- FLT3-* | 90 | NA | F | 26 |
| 518 | M7 | t(3,11) | *NPM1- FLT3-* | 36 | Intermediate | F | 79 |

**Table S2 Diagnosis and characteristics of HD study cohort**

| **Laboratory code** | **BM origin sample** | **Sex** | **Age** |
| --- | --- | --- | --- |
| 498 | BM aspiration | F | 45 |
| 442 | BM aspiration | F | 62 |
| 564 | BM –hip replacement | F | 48 |
| 557 | BM –hip replacement | F | 60 |
| 40883 | Lymphoma with no BM intervention | M | 40 |
| 40604 | Lymphoma with no BM intervention | M | 47 |
| 40754 | Lymphoma with no BM intervention | M | 82 |
| 40582 | Lymphoma with no BM intervention | M | 65 |
| 40373 | Lymphoma with no BM intervention | F | 58 |
| 40513 | Lymphoma with no BM intervention | F | 80 |
| 569 | BM –hip replacement | F | 63 |
| 538 | BM –hip replacement | F | 43 |
| 544 | BM –hip replacement | F | 50 |
| 507 | BM –hip replacement | F | 42 |
| 529 | BM –hip replacement | F | 79 |
| 550 | BM –hip replacement | M | 65 |
| 534 | BM –hip replacement | F | 75 |
| 558 | BM –hip replacement | F | 37 |
| 484 | BM aspiration | M | 18 |
| hMSC | Lonza |  |  |
| 486 | BM aspiration | M | 18 |
| 517 | BM –hip replacement | M | 67 |
| 510 | BM –hip replacement | F | 71 |
| 571 | BM –hip replacement | F | 50 |
| 570 | BM –hip replacement | M | 27 |
| 546 | BM –hip replacement | F | 28 |

**Table S3 Antibodies used in the study**

| **Antibody** | **Fluorophore** | **Manufacturer** | **Application** |
| --- | --- | --- | --- |
| CD45 | BUV395 | BD Biosciences 563792 | Flow cytometry |
| CD33 | BV510 | BD Biosciences #563257 |
| CD38 | FITC | BioLegend #303510 |
| CD73 | BV605 | BD Biosciences #563199 |
| CD90 | APC | BioLegend #328114 |
| CD14 | PE | BioLegend #325606 |

**Table S4 Genetic alterations**

| **#Patient** | **Chr** | **Position (bp)** | **Ref nucleotide** | **Variant** | **Gene** | **VAF** | **AA Subs** | **Type of change** | **Polyphen2_HVAR_score** | **Polyphen2_HVAR_pred** |
| --- | --- | --- | --- | --- | --- | --- | --- | --- | --- | --- |
| 1 | 1 | 152303806 | C | G | *FLG* | 0.1 | E3694Q | Missense | 0.883 | Possibly damaging |
| 2 | 1 | 152304223 | A | G | *FLG* | 0.1 | W3555R | Missense | 0.118 | Benign |
| 3 | 1 | 152304285 | T | C | *FLG* | 0.12 | N3534S | Missense | 0.796 | Possibly damaging |
|  | 1 | 152304286 | T | A | *FLG* | 0.12 | N3534Y | Missense | 0.857 | Possibly damaging |
|  | 1 | 152307871 | C | T | *FLG* | 0.11 | D2339N | Missense | 0.942 | Probably damaging |
|  | 1 | 152309616 | T | C | *FLG* | 0.1 | E1757G | Missense | 0.082 | Benign |
| 4 | 1 | 152308083 | T | C | *FLG* | 0.11 | H2268R | Missense | 0.001 | Benign |
| 6 | 1 | 152312002 | A | G | *FLG* | 0.07 | W962R | Missense | 0 | Benign |
|  | 1 | 152312029 | C | G | *FLG* | 0.08 | E953Q | Missense | 0.885 | Possibly damaging |
| 1 | 1 | 201224749 | C | T | *IGFN1* | 0.26 | T3454I | Missense | 0.125 | Benign |
| 6 | 1 | 201209922 | A | G | *IGFN1* | 0.09 | N1677D | Missense | No data | No data |
| 5 | 1 | 201211012 | C | T | *IGFN1* | 0.23 | A2040V | Missense | No data | No data |
| 3 | 1 | 201211017 | G | A | *IGFN1* | 0.33 | E2042K | Missense | No data | No data |
|  | 1 | 201211020 | A | G | *IGFN1* | 0.27 | R2043G | Missense | No data | No data |
|  | 1 | 201211025 | A | G | *IGFN1* | 0.22 | I2044M | Missense | No data | No data |
|  | 1 | 201211044 | G | A | *IGFN1* | 0.1 | G2051S | Missense | No data | No data |
| 2 | 1 | 201209640 | A | T | *IGFN1* | 0.19 | S1583C | Missense | No data | No data |
| 6 | 3 | 75738311 | G | A | *ZNF717* | 0.14 | L438F | Missense | 0.993 | Probably damaging |
|  | 3 | 75738323 | G | A | *ZNF717* | 0.12 | H434Y | Missense | 0.66 | Possibly damaging |
|  | 3 | 75738325 | C | A | *ZNF717* | 0.11 | S433I | Missense | 0.873 | Possibly damaging |
|  | 3 | 75738360 | C | A | *ZNF717* | 0.11 | K421N | Missense | 0.992 | Probably damaging |
| 1 | 3 | 75738979 | C | T | *ZNF717* | 0.07 | G215E | Missense | 0.249 | Benign |
| 7 | 3 | 75738360 | C | A | *ZNF717* | 0.1 | K421N | Missense | 0.992 | Probably damaging |
| 3 | 3 | 75738118 | G | A | *ZNF717* | 0.07 | T502I | Missense | 0.989 | Probably damaging |
| 2 | 3 | 75738574 | C | T | *ZNF717* | 0.08 | R350H | Missense | 0.332 | Benign |
| 3 | 14 | 104944046 | T | C | *AHNAK2* | 0.14 | K3802R | Missense | 0.471 | Possibly damaging |
| 3 | 14 | 104944124 | G | C | *AHNAK2* | 0.15 | P3776R | Missense | 0.944 | Probably damaging |
| 5 | 14 | 104945826 | C | G | *AHNAK2* | 0.11 | V3209L | Missense | 0.007 | Benign |
| 1 | 14 | 104949045 | G | C | *AHNAK2* | 0.08 | Q2136E | Missense | 0.002 | Benign |
| 1 | 14 | 104949049 | A | C | *AHNAK2* | 0.07 | H2134Q | Missense | 0 | Benign |
| 7 | 14 | 104946703 | T | C | *AHNAK2* | 0.07 | I2916M | Missense | 0.006 | Benign |
| 7 | 14 | 104946705 | T | C | *AHNAK2* | 0.07 | I2916V | Missense | 0 | Benign |
| 7 | 14 | 104949074 | G | C | *AHNAK2* | 0.07 | P2126R | Missense | 0.261 | Benign |
| 7 | 14 | 104949094 | C | G | *AHNAK2* | 0.08 | M2119I | Missense | 0.01 | Benign |
| 7 | 14 | 104949096 | T | C | *AHNAK2* | 0.08 | M2119V | Missense | 0.002 | Benign |
| 4 | 19 | 39893949 | C | T | *FCGBP* | 0.13 | E1938K | Missense | 0.34 | Benign |
| 5 | 19 | 39893949 | C | T | *FCGBP* | 0.12 | E1938K | Missense | 0.34 | Benign |
| 6 | 19 | 39885791 | A | G | *FCGBP* | 0.08 | L2757P | Missense | 0.001 | Benign |
| 6 | 19 | 39902034 | G | T | *FCGBP* | 0.08 | P1559Q | Missense | 0.759 | Possibly damaging |
| 6 | 19 | 39902043 | A | G | *FCGBP* | 0.11 | L1556P | Missense | 0.028 | Benign |
| 7 | 19 | 39893180 | C | T | *FCGBP* | 0.1 | G2063S | Missense | 0.956 | Probably damaging |

Chr: chromosome #; Ref nucleotide: reference nucleotide; VAF: variant allele frequency; AA Subs: amino acid substitutions

**Table S5 Primers used for sequencing**

|  | **Forward primer (5`->3`)** | **Reverse primer (5`->3`)** |
| --- | --- | --- |
| ***AHNAK2- a*** | GAGAAGGAGGACACGGATGTTGC | CCCCGCTTGCTCTTTATGGATTG |
| ***β2m*** | GAGGCTATCCAGCGTACTCCA | CGGCAGGCATACTCATCTTTT |
| ***FABP4*** | TGGTTGATTTTCCATCCCAT | TACTGGGCCAGGAATTTGAC |
| ***PPARᵞ1*** | CGTGGCCGCAGATTTGAA | CTTCCATTACGGAGAGATCCAC |
| ***H3A*** | AAGCAGACTGCCCGCAAAT | GGCCTGTAACGATGAGGTTTC |
| ***ADRα1A*** | AGAAGAAAGCGGCCAAAACG | TGGAGCATGGGTATATGATGGG |
